# Ophthalmology of clinically normal alpacas (*Vicugna pacos*) in the United Kingdom: a cross-sectional study

**DOI:** 10.1136/vr.105758

**Published:** 2020-04-17

**Authors:** Josephine Faulkner, David Leonard Williams, Karin Mueller

**Affiliations:** 1 Department of Veterinary Medicine, Cambridge University, Cambridge, UK; 2 Faculty of Veterinary Medicine, Ghent University, Merelbeke, Belgium; 3 School of Veterinary Science, University of Liverpool, Liverpool, Merseyside, UK

**Keywords:** Ophthalmology, Eyes, Camelids, Alpacas

## Abstract

**Background:**

Alpacas are being more frequently presented to veterinarians in the UK. It is important to validate whether published normal ocular parameters are consistent with the alpaca population in the UK.

**Methods:**

Ophthalmic examinations were performed on healthy alpacas (*Vicugna pacos*) from three farms in East Anglia, UK.

**Results:**

On direct ophthalmoscopy of 35 alpacas, there was a 50 per cent prevalence of opacities within the lens in alpacas older than two years old (n=8/16). There was a 36.8 per cent prevalence of persistent hyaloid arteries in alpacas under two years old (n=7/19). The mean Schirmer tear test-1 value was 20.0 ±6 mm/minute (n=40). The mean intraocular pressure measured by rebound tonometry was 17.2 ±5.5 mmHg (n=46), and applanation tonometry resulted in statistically similar values (P=0.30; n=25). There was a significant variation in intraocular pressure throughout a 24-hour period (n=8). Fluorescein dye was not detected at the nostrils of any of the alpacas which underwent a Jones test to assess nasolacrimal duct patency (n=8).

**Conclusion:**

The ophthalmic findings appear largely consistent with previously published values from North America and continental Europe. Variations include the large range of measurements obtained and evidence of diurnal variation in intraocular pressure.

## Introduction

Alpaca eyes are protuberant and susceptible to trauma.^1–5^ With the increase in population of alpacas in the UK, these animals are being more frequently presented to veterinarians for assessment and treatment. The diagnosis of clinical disease relies on a sound understanding of typical appearance and normal parameters, as well as their variations. All published details of measurement of ocular parameters and variations in clinically healthy alpacas are from the USA, Canada and Italy. It is important to validate whether these findings are consistent in the alpaca population in the UK.

Developmental and heritable eye disease has been reported in alpacas.^6 7^ Importantly, some developmental variations in young animals, such as persistent hyaloid artery and Bergmeister’s papillae, appear to be of no clinical significance.^4^ A relationship between coat colour, iris colour and fundus pigmentation has been suggested.^8^ There is also a suggestion (but not yet proven) that coat or iris colour is related to congenital deafness, with less pigmented individuals more likely to have congenital deafness.^9^ Incidental detection of abnormalities on ophthalmoscopic examination is commonly reported, with some abnormalities likely a result of previous trauma, including superficial corneal scars, anterior and posterior synechiae, iris to iris persistent pupillary membranes, cataracts, subluxated lenses, and vitreous opacities.^8 9^

### Schirmer tear test

The Schirmer tear test without local anaesthetic application (STT-1) is used to test tear production. In alpacas there are reported mean±sd values of 20.88±4.04 mm/minute (range 15.50–30.50)^10^ and 16.9±4.1 mm/minute (range 12–23).^11^

### Intraocular pressure

Intraocular pressure (IOP) is useful in the diagnosis and monitoring of glaucoma, uveitis and trauma. Mean results from several studies on applanation tonometry range from 12.51 mmHg to 19 mmHg.^9 11–14^ Rebound tonometry of one population showed a mean result of 14.21 mmHg.^12^ Time of day is reported not to have a significant effect on IOP.^13^

### Nasolacrimal duct patency

Nasolacrimal duct obstruction has been reported.^15 16^ However the authors are not aware of any publication on using fluorescein dye to check for nasolacrimal duct patency (Jones test) in alpacas and the normal time for dye to appear at the nostril.

## Materials and methods

Fifty-one alpacas of both sexes and fleece phenotypes, and of various ages, were recruited from three farms in East Anglia between July and September 2010. All were presented as healthy by the owners and considered clinically normal following brief physical examination by the authors. Farm, age, sex, fleece phenotype and fleece colour were recorded for each animal. A range of examinations were performed for each alpaca depending on equipment available on the day and the tolerance of the alpaca to examination. Both eyes were assessed in all tests unless the animal became stressed by the procedure.

Alpacas used in the 24-hour IOP study were housed and monitored in pens at the Queen’s Veterinary School Hospital Farm Animal Unit. All other examinations were performed in the alpacas’ home environment, in the corner of a barn during the afternoon.

They were allowed to lower into sternal recumbency (a cushed position) if preferred, but the majority tolerated examination standing with loose manual restraint by neck or bracelet hold, with care taken to avoid compression of the jugular vein. The alpacas were accustomed to being handled and chemical restraint was not used.

### Ophthalmoscopy

Thirty-five alpacas were examined with both direct ophthalmoscopy (Keeler Practitioner, Keeler, Windsor, UK) and with slit lamp biomicroscopy (Hawk Eye Portable Slit Lamp, Dioptrix, Toulouse, France). A drape was placed over the head of the alpaca and the veterinarian as necessary to achieve dilation of the pupil sufficient for examination of the lens and fundus without chemical mydriasis. Appearance and variation of the structures of the eye were recorded.

### Schirmer tear test

Tear production was measured in 40 alpacas using Schirmer tear test strips (Schering-Plough Animal Health) without prior application of local anaesthetic (STT-1). The 35-mm strips were placed in the lower lateral conjunctival fornix for 60 seconds with the eye gently held closed. Measurements were read immediately at 60 seconds and recorded as mm/minute. A 2 x 2 cross-over study (n=4) was initially performed to establish whether there was a significant difference in the results between using the upper and lower lids.

### Intraocular pressure

IOP was estimated from the central cornea in 46 alpacas using the TonoVet rebound tonometer (Icare VET Finland Oy, Vantaa, Finland). The tonometer was set to the ‘horse’ manufacturer setting as has been done in another study.^12^ Six measurements were acquired from each eye to obtain the final reading as the average of four measurements, excluding the highest and lowest result. A pilot study was performed on eight alpacas in a 4 x 4 cross-over study to establish whether two different holding techniques affected IOP measured on the rebound tonometer.

In a subset of 25 alpacas in this group, the Tono-Pen XL applanation tonometer (Reichert Technologies, Depew, New York, USA) was also used following the TonoVet. Two to three drops of topical local anaesthetic (Minims Tetracaine Hydrochloride 1.0% w/v, Bausch & Lomb UK) were applied to the cornea about three minutes before the Tono-Pen was used. The mean of four valid applanation readings was recorded for each alpaca.

Eight female yearling alpacas were used for 24-hour IOP monitoring. After an overnight acclimatisation period, rebound tonometry was performed bilaterally every three hours, from 09.00 to 06.00 the following day.

### Nasolacrimal duct patency

The use of fluorescein dye to assess nasolacrimal duct patency was assessed unilaterally in four yearling females and bilaterally in four adult males. A fluorescein sodium ophthalmic strip (Fluorets, Chauvin Pharmaceuticals) was dipped in saline solution and stroked across the lower bulbar conjunctiva. Blinking allowed the dye to spread throughout the eye. Alpacas were assessed initially up to 39 minutes after dye application, then again one hour later, for the presence of dye at the nostrils.

### Statistical analysis

Data analysis was performed with Microsoft Excel (Microsoft Office V.15.0.5101.1002, Redmond, Washington, USA). Results are presented as mean±sd (range). Statistical significance of the differences related to instrument, technique, laterality or sex was determined using a two-tailed Student’s paired *t* test. Probability values of P≤0.05 were considered statistically significant. For analysis of associations with age, alpacas were grouped into juveniles (<30 days old), yearlings (11–15 months), and adult group 1 (2–8 years old) and group 2 (≥8 years old), and single-factor analysis of variance (ANOVA) was used to determine statistical significance of differences between groups. ANOVA was also used for analysis of diurnal variation in IOP.

## Results

Eighteen males and 33 females were included in the study. Fleece phenotype consisted of 46 Huacaya and six Suri. Age groups included juveniles (n=9; 9–26 days old), yearlings (n=11; 11–15 months old) and adults (n=31; 2–16.9 years old). The signalment and range of examinations performed for each alpaca are presented in table 1.

**Table 1:** Alpaca signalment and ophthalmic examinations performed (N=51)

Identification	Farm	Age*	Sex	Fleece phenotype	Fleece colour	Examination performed					
						Ophthalmoscopy	STT	IOP TonoVet	IOP Tono-Pen	IOP 24 hours	NLD
1	A	9 d	F	Suri	White	·	·	·			
2	A	11 d	F	Huacaya	White	·	·	·			
3	A	11 d	M	Huacaya	White	·	·	·			
4	A	16 d	M	Huacaya	Fawn	·	·	·			
5	A	18 d	M	Huacaya	Fawn	·	·	·			
6	A	23 d	M	Huacaya	White	·	·	·			
7	A	23 d	M	Huacaya	White	·	·	·			
8	A	26 d	F	Huacaya	White	·	·	·			
9	A	26 d	M	Huacaya	White	·	·	·			
10	A	10.6 m	F	Huacaya	White	·	·	·	·	·	
11	A	11.1 m	F	Huacaya	White	·	·	·	·	·	
12	A	11.3 m	F	Huacaya	White	·	·	·	·	·	·
13	A	12.2 m	F	Huacaya	White	·	·	·	·	·	
14	A	12.4 m	F	Huacaya	White		·	·		·	
15	A	12.5 m	F	Huacaya	White	·	·	·	·	·	·
16	A	12.7 m	F	Huacaya	Brown	·	·	·	·	·	·
17	A	12.8 m	F	Huacaya	White	·	·	·	·	·	·
18	A	13.1 m	F	Huacaya	Brown	·	·	·	·		
19	A	13.2 m	F	Huacaya	White	·	·	·	·		
20	A	14.5 m	F	Huacaya	White	·		·	·		
21	B	2 y	M	Huacaya	White		·				
22	B	2.3 y	M	Huacaya	White		·				
23	A	3.1 y	F	Huacaya	White	·	·	·			
24	A	3.1 y	F	Suri	Brown	·	·	·			
25	A	4 y	M	Huacaya	White	·	·	·	·		
26	C	3.9 y	F	Huacaya	White			·	·		
27	B	4.1 y	M	Huacaya	White		·				
28	B	4.1 y	M	Huacaya	White		·				
29	C	4.1 y	M	Suri	White			·	·		·
30	A	4.2 y	F	Huacaya	White	·	·	·			
31	A	4.5 y	F	Huacaya	White	·	·	·			
32	C	5.3 y	M	Huacaya	Fawn			·	·		·
33	C	5.4 y	M	Huacaya	White			·	·		·
34	C	6.1 y	M	Huacaya	White			·	·		
35	C	6.3 y	F	Suri	Fawn			·	·		
36	A	6.6 y	F	Huacaya	Fawn	·	·	·			
37	A	6.9 y	F	Huacaya	White	·	·	·	·		
38	C	7.4 y	M	Huacaya	Multi†			·	·		·
39	A	7.5 y	F	Huacaya	White	·	·	·			
40	B	7.5 y	M	Huacaya	White		·				
41	C	7.7 y	F	Suri	White			·	·		
42	A	8 y	M	Huacaya	Brown	·	·	·	·		
43	C	8.4 y	F	Huacaya	White			·	·		
44	C	8.4 y	F	Huacaya	Brown			·	·		
45	A	8.5 y	F	Huacaya	White	·	·	·			
46	A	9.3 y	F	Huacaya	White	·	·	·	·		
47	A	9.8 y	F	Suri	Brown	·	·	·			
48	A	10.5 y	F	Huacaya	White	·	·	·			
49	A	12.9 y	F	Huacaya	Brown	·	·	·	·		
50	A	13.5 y	F	Huacaya	White	·	·	·			
51	A	16.9 y	F	Huacaya	Fawn	·	·	·			
					Total	35	40	46	25	8	8

*In days (d), months (m) and years (y).

†Black, white, silver.

F, female; IOP, intraocular pressure; M, male; NLD, nasolacrimal duct; STT, Schirmer tear test.

### Ophthalmoscopy

Seventy eyes of 35 alpacas (eight males, 27 females) were examined. Twenty-five alpacas had a white fleece, with brown (n=3), grey (n=21) and blue (n=1) irides. Six alpacas had a brown fleece, all with brown irides. Four alpacas were fawn with brown (n=1) and grey (n=3) irides. The majority of alpacas (n=33) had slate grey/brown fundi. Pink/non-pigmented regions in the fundus were seen in a 1.2-year-old white alpaca with blue irides and a one-year-old white alpaca with grey irides. Coloration and pigmentation findings are presented in table 2.

**Table 2:** Comparison of fleece, iris and fundus coloration and pigmentation in 35 alpacas

Fleece colour	n	Iris colour	n	Pigmented (grey/brown) fundus	Non-pigmented (pink) fundus (fully or partially)
White	25	Brown	3	3	0
		Grey	21	20	1
		Blue	1	0	1
Brown	6	Brown	6	6	0
Fawn	4	Brown	1	1	0
		Grey	3	3	0

Lens opacity prevalence was 37.5 per cent (n=3/8) in young adults (two to eight years old), with all affected alpacas at least four years old. There was a 62.5 per cent prevalence (n=5/8) of lens opacities in alpacas over the age of eight years. Easily discernible lens changes included nuclear sclerosis (n=1) and immature cataract (n=6). Of these seven alpacas, three had concurrent subtle multifocal opacities visible in the lens: dots (n=2) and lines (n=1). One alpaca displayed subtle multifocal opaque dots without other changes.

In the posterior chamber, juvenile alpacas had a 33.3 per cent prevalence (n=3/9) of persistent hyaloid arteries, all present bilaterally. The prevalence in yearling alpacas was 40 per cent (n=4/10), two bilaterally and two unilaterally. No persistent hyaloid arteries were detected in any adult alpacas. Comparison of lens and posterior chamber variations and abnormalities within age groups is presented in table 3.

**Table 3:** Lens and posterior chamber variations and abnormalities in 35 alpacas (by age group)

Age	Alpacas examined (n)	Persistent hyaloid artery	Lens opacity
<30 d	9	33.3% (n=3)*	0
11–15 m	10	40% (n=4)†	0
2–8 y	8	0	37.5% (n=3)
≥8 y	8	0	62.5% (n=5)

*All bilateral.

†2 unilateral, 2 bilateral.

d, days old; m, months old; y, years old.

### Schirmer tear test

In an initial 2 x 2 cross-over pilot study (n=4), there was no significant difference in the results between using the upper and lower lids (P=0.10). Placement into the lower lid was chosen as the easier method for the operator.

Forty alpacas (13 males, 27 females) underwent STT-1. Data from 74 out of 80 eyes were collected as there was poor tolerance to the procedure in the second eye in two alpacas, and in four eyes the strip did not remain in the eye for the whole 60 seconds. In two eyes, the tears reached the end of the 35-mm strip shortly before 60 seconds, and during analysis of the results these measurements were entered as 40 mm/minute.

The mean STT-1 was 20.0±6.0 mm/minute (range 10–40). There was no significant difference between sexes (P=0.23) or age groups (ANOVA: F=1.21, Fcrit=2.74, F<Fcrit). There was a statistically significant difference between the left and right eyes (P=0.04). The mean for the left was 21.5±6.3 mm/minute oculus sinister (OS), and for the right 18.6±5.5 mm/minute oculus dextrus (OD).

### Intraocular pressure

There was no significant difference in IOP measured on the rebound tonometer between the neck hold and the bracelet hold (P=0.896) in the 4 x 4 cross-over study, and therefore either hold was used in the main study according to handler preference.

IOP was estimated with rebound tonometry in the 92 eyes of 46 alpacas (13 males, 33 females). The mean IOP was 17.2±5.5 mmHg (range 5–38). There were no significant differences between the left and right eyes (P=0.32), male and female (P=0.31), or age (ANOVA: F=2.23, Fcrit=2.71, F<Fcrit).

The subgroup that had both rebound and applanation tonometry performed consisted of 25 alpacas (seven males, 18 females). In 50 eyes there was no significant difference between the two measuring methods (P=0.30). The mean IOP with rebound tonometry was 16.4±5.3 mmHg (range 5–29). The mean IOP with applanation tonometry was 17.6±5.7 mmHg (range 8–36).

**Figure 1:**
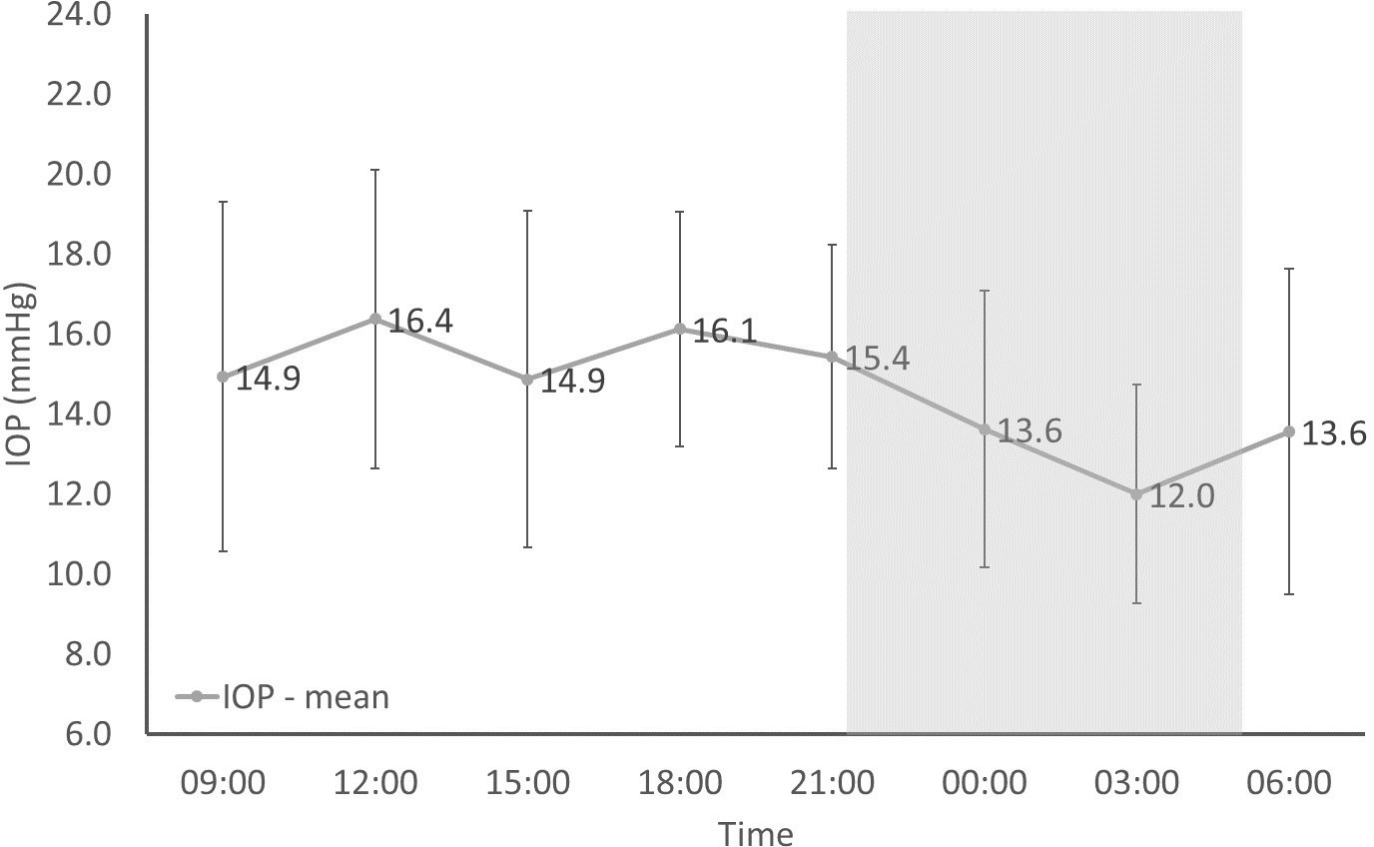
Variation in mean intraocular pressure (IOP) over a 24-hour period in eight female yearling alpacas (16 eyes) using a TonoVet rebound tonometer. Error bars show 1 sd. Grey shading indicates the time between sunset and sunrise at the time of the study in July.

The results for the circadian rhythm for IOP are shown in figure 1. There was a significant difference between the means at each time point (ANOVA: Fcrit=2.09, F=2.69, F>Fcrit).

### Nasolacrimal duct patency

No fluorescein dye appeared at the nostrils for any of the eight alpacas tested.

## Discussion

All procedures were well tolerated by the majority of alpacas, with the only objection seen by two alpacas to the STT-1. The TonoVet was found to be quicker to use than the Tono-Pen as it was tolerated better by the alpacas, and had the added advantage of not requiring topical local anaesthesia. There were no reactions to the solutions administered to the eye and no damage to the eye was observed as a result of the procedures. Manual restraint using one handler was sufficient and the use of penned areas in the barns was acceptable for all procedures.

### Ophthalmoscopy

The proposed association between coat colour, iris and fundus colour^8^ is supported to some degree within this population. The only blue-eyed white alpaca examined had non-pigmented regions in the fundus, as did one white alpaca with grey irides. The remainder of the alpacas had grey/brown pigmented fundi, whether they were white, fawn or brown fleeced alpacas with grey or brown irides. If coat or iris colour is genetically related to congenital deafness, then ophthalmoscopy may be a useful initial screening tool; however, this association still needs to be determined.^9^ None of the alpacas in the study was reported to display signs of deafness; however, this may be difficult to pick up in alpacas in a herd situation.

The oldest alpaca with persistent hyaloid arteries in this study was 13 months old. Given the fairly high prevalence (36.8 per cent; n=7/19) in young alpacas, persistent hyaloid arteries appear to be a normal developmental variation. It is unknown whether there are any consequences (positive or negative) of the delayed closure of patency; however, it is useful to be aware of their possible presence during examination or where cataract surgery is to be attempted.^17^

No hereditary lens abnormalities such as juvenile cataract were seen in young alpacas. About half the mature animals showed lens changes, including nuclear sclerosis, cataract and other opacities. It is possible that some of the more subtle dot and line opacities in the posterior pole of the lens at the site of Mittendorf dot may have resulted from persistent hyaloid vasculature.^18^

### Schirmer tear test

STT-1 is used to test tear production, and values are altered in keratoconjunctivitis sicca and surface tear film disorders. The mean STT-1 value of 20.0±6.0 mm/minute agrees well with the previously published value of 20.88±4.04 mm/minute,^10^ while slightly less well with another published value of 16.9±4.1 mm/minute.^11^ The range of values obtained here (10–40 mm/minute) and the sd are wider than in other publications (ranges 15.5–30.5 mm/minute^10^ and 12–23 mm/minute^11^). The eyelids were held closed gently in this study to ensure strip retention, and this may have elevated some values. Regarding technique, the small cross-over study performed showed no significant difference between using the upper and lower eyelids for placement of the STT strip. Both upper^10^ and lower^11^ conjunctival fornices have been used in previous studies, and it has been determined that tear strip position for STT-1 in horses is insignificant.^19^

No difference in STT value was found between age groups. This is consistent with one study^11^ but differs from another,^10^ whose authors found STT-1 to increase by 3.45 mm/minute for every 10-year increase in age. The reason for the statistically significant difference (P=0.015) in means between left and right eye STT remains unexplained; however, the difference in values are not considered clinically important. The order in which eyes were examined was not controlled or formally randomised, so there may have been some bias in order, affecting the results between the left and right eyes. The contralateral (second) eye may have been stimulated to also increase tear production while the first eye underwent the STT-1, resulting in a higher value when the second eye underwent the STT-1. The authors are cautious of over-reporting this finding (in case of a type I error).

### Intraocular pressure

IOP is useful in the diagnosis and monitoring of glaucoma, uveitis and trauma. A comparison of IOP results with previously published values is shown in table 4. Means from this study for both applanation and rebound tonometry are generally higher than others’ findings, with a wider sd and range.

**Table 4:** Comparison of IOP results in alpacas with previously published values

	Eyes (N)	IOP, mmHg±sd (range)	
		Applanation tonometry (Tono-Pen)	Rebound tonometry (TonoVet)
Results	92		17.2±5.4 (5–38)
	50	17.6±5.7 (8–36)	16.4±5.3 (5–29)
McDonald *et al* 12	80	12.51±2.78 (6.00–19.33)	14.21±2.73 (8.67–20.67)
Pietro *et al* 11	46	15.3±1.8 (13–18)	
Webb *et al* 9	46	19±4 (n/a)	
Nuhsbaum *et al* 14	20	16.14±3.74 (n/a)	
Willis *et al* 13	36	14.85±0.45 (11–21)	

IOP, intraocular pressure; n/a, not available.

No significant sex-related or age-related change in IOP was found, in agreement with most other studies but in discrepancy with one.^14^

Factors which could possibly cause discrepancies between the studies may include population differences in corneal thickness and curvature,^20 21^ instrument technique and calibration, undetected pathology, stress, different pressure on vasculature in the neck, and choice of topical local anaesthetics.^22^

No statistically significant difference was found between measurements obtained by rebound and applanation tonometry, in contrast to one previous study^12^ in which IOP was significantly higher (P=0.002) for rebound tonometry compared with applanation tonometer; however, the authors concluded that the difference (1.70 mmHg) was not clinically significant.

The 24-hour IOP data showed a significant difference between the means at each time and indications of a circadian trend with a sine-like distribution with time. Higher values were obtained during the daytime (from 09.00 to 21.00) and lower measurements during the dark night-time period and early morning (from 12.00 to 06.00). IOP circadian rhythms have been recorded in rabbits,^23^ cats,^24^ dogs^25^ and horses,^26^ and so it is feasible for alpacas to have the same. One previous study found that the time of measurement had no significant effect on IOP in camelids, however it did not perform any measurements between 19.00 and 07.00.^13^ The reported IOP was lower at 19.00 but not significantly so.

### Nasolacrimal duct patency

The findings of the present study suggest that the fluorescein dye Jones test for nasolacrimal duct patency may not be of clinical use in alpacas as none of the subjects displayed fluorescein stain at the nostrils within one to two hours. Although the alpacas in this study were not further clinically evaluated for nasolacrimal patency, it is unlikely that all alpacas in the tested group would be affected by a congenital or mechanical obstruction as they displayed no clinical signs. The use of the Jones test should be further investigated in alpacas, with monitoring continued over time periods longer than the one to two hours in this study and with further examination of the anatomy of the nasolacrimal duct.

### Limitations

The study used convenience sampling, recruiting three farms in the same region of the UK within easy travelling distance. Not all procedures were performed on all alpacas, so the order in which procedures were performed was not consistent. Examinations were performed by one of two veterinarians and a veterinary student following a brief training period; therefore, expertise level was variable and consistency in technique may have varied.

## Conclusion

The findings of this study should be taken into account alongside previous publications when examining and assessing alpaca eyes as measurements and observations may influence prepurchase examination conclusions, clinical monitoring of disease and decision-making.

The findings appear consistent with previously published values, but there are some variations identified, including the range of measurements obtained and evidence of diurnal circadian rhythm in IOP. From a practical point, the cross-over studies suggest that there is no difference between lower or upper eyelid for STT-1 or between neck hold or bracelet hold for restraint for IOP measurements. The Jones test did not appear to be a valid test for nasolacrimal duct patency in alpacas; however, this should be further investigated.
